# The effects of tissue fixation on sequencing and transcript abundance of nucleic acids from microdissected liver samples of smallmouth bass (*Micropterus dolomieu*)

**DOI:** 10.1371/journal.pone.0236104

**Published:** 2020-08-10

**Authors:** Heather L. Walsh, Adam J. Sperry, Vicki S. Blazer

**Affiliations:** U.S. Geological Survey, National Fish Health Research Laboratory, Leetown Science Center, Kearneysville, West Virginia, United States of America; National Institute of Child Health and Human Development (NICHD), NIH, UNITED STATES

## Abstract

There is an increasing emphasis on effects-based monitoring to document responses associated with exposure to complex mixtures of chemicals, climate change, pathogens, parasites and other environmental stressors in fish populations. For decades aquatic monitoring programs have included the collection of tissues preserved for microscopic pathology. Consequently, formalin-fixed, paraffin-embedded (FFPE) tissue can be an important reservoir of nucleic acids as technologies emerge that utilize molecular endpoints. Despite the cross-linking effects of formalin, its impact on nucleic acid quality and concentration, amplification, and sequencing are not well described. While fresh-frozen tissue is optimal for working with nucleic acids, FFPE samples have been shown to be conducive for molecular studies. Laser capture microdissection (LCM) is one technology which allows for collection of specific regions or cell populations from fresh or preserved specimens with pathological alterations, pathogens, or parasites. In this study, smallmouth bass (*Micropterus dolomieu*) liver was preserved in three different fixatives, including 10% neutral buffered formalin (NBF), Z-Fix® (ZF), and PAXgene® (PG) for four time periods (24 hr, 48 hr, seven days, and 14 days). Controls consisted of pieces of liver preserved in RNALater® or 95% ethanol. Smallmouth bass were chosen as they are an economically important sportfish and have been utilized as indicators of exposure to endocrine disruptors and other environmental stressors. Small liver sections were cut out with laser microdissection and DNA and RNA were purified and analyzed for nucleic acid concentration and quality. Sanger sequencing and the NanoString nCounter® technology were used to assess the suitability of these samples in downstream molecular techniques. The results revealed that of the formalin fixatives, NBF samples fixed for 24 and 48 hr were superior to ZF samples for both Sanger sequencing and the Nanostring nCounter®. The non-formalin PAXgene® samples were equally successful and they showed greater stability in nucleic acid quality and concentration over longer fixation times. This study demonstrated that small quantities of preserved tissue from smallmouth bass can be utilized in downstream molecular techniques; however, future studies will need to optimize the methods presented here for different tissue types, fish species, and pathological conditions.

## Introduction

Globally, environmental monitoring programs are increasingly used to identify adverse effects of human activities on aquatic resources [1–4]. The recognition that there are numerous chemical contaminants, environmental stressors, as well as new and emerging pathogens/parasites co-occurring has led to an emphasis on biological environmental effects-based assessments utilizing resident indicator fish species or caged model species [5–7]. Histopathology has been used for many years to assess the health of wild fishes both for specific studies as well as part of effects-based monitoring programs [8–11]. More recently, genomic endpoints are also being incorporated into environmental monitoring and risk assessment [12–14]. When both histopathology and molecular analyses are part of an assessment, pieces of a tissue are commonly preserved in 10% buffered formalin or a similar preservative and adjacent, separate pieces are preserved in RNAlater®, 95% ethanol, or frozen for molecular analyses [15–17]. However, for alterations not visible, the tissue piece chosen for gene expression may not contain the same cellular components or alterations as those within the histology section.

The use of formalin fixed, paraffin-embedded (FFPE) tissue has been regarded as a valuable reservoir of preserved nucleic acids in mammalian studies [18–23]. Although FFPE tissues provide a vast source of pathologically diverse types of genetic material, there are drawbacks compared to other tissue preservation methods. Formalin fixation causes nucleic acids to fragment, degrade, and cross-link [24]. Frozen tissues or tissues specifically preserved for downstream nucleic acid applications do not experience the type of degradation observed from formalin fixation. Despite these setbacks, nucleic acids extracted from FFPE tissue have proven to be suitable for use in end-point PCR [25], real-time qPCR [26, 27], and Next-generation sequencing [28, 29]. Optimization of FFPE tissues for downstream nucleic acid applications has been attempted in multiple studies by evaluation of different fixation methods [30, 31], tissue handling and processing times [32, 33], and extraction methods [23, 25, 34, 35].

Laser capture microdissection (LCM) utilizes a microscope equipped with a laser to target and isolate specific cells from a heterogeneous population of cells [36]. Single cells, foci of cell populations within a tissue, or pathogens and parasites can be microdissected. Hence, nucleic acids from specific cell populations of interest can be analyzed for gene expression studies, transcriptome development, or molecular identification of pathogens and parasites. This allows for a more direct connection between the histopathology and molecular analyses. LCM has been previously utilized in fish-related studies [15, 16, 37–40] with frozen sections. Snap-frozen tissue is optimal for use with LCM for the downstream recovery of nucleic acids. However, the use of snap-frozen tissue is not always feasible, particularly in wild fish studies where removal and fixation of the organs occurs in the field and it can be days before tissues are returned to the laboratory and processed. LCM of FFPE tissue can bridge the gap between microscopy and molecular analyses [41]. As with other species, there is a vast amount of archival FFPE (or similarly preserved) fish tissue that could be useful for molecular analyses.

The aim of this study was to determine how fixative type and fixation time affects nucleic acids in FFPE smallmouth bass liver tissue dissected with LCM. Smallmouth bass (*Micropterus dolomieu*) are utilized in ongoing monitoring and assessment studies as an indicator species of exposure to endocrine-disrupting and other contaminants. Additionally, they are a non-model, but economically important, species. To address the utility of paraffin-embedded fish tissue for molecular studies, smallmouth bass liver was sampled and preserved for four time periods (24 hr, 48 hr, seven days, and 14 days) in 10% neutral buffered formalin (NBF), Z-Fix® (ZF), and the non-formalin fixative PAXgene® (PG). The PAXgene® Tissue System, was designed to improve tissue quality for parallel molecular and morphological analyses [42]. Similarly, ZF (a zinc-based formalin solution) was chosen as it has been shown to produce higher yields of DNA and RNA when compared to samples fixed in NBF [43]. In addition to DNA and RNA quantification, downstream molecular techniques, including Sanger sequencing and the Nanostring nCounter® digital multiplexed gene expression assay [44], were used to determine if nucleic acids extracted from LCM tissue sections would have utility in future studies. To the best of our knowledge, this study provides novel research on the optimization of fixative type and fixation time for the use of fish tissue extracts with Nanostring nCounter® technology.

## Materials and methods

### Ethics statement and smallmouth bass sample collection

All procedures, including the handling and euthanasia of fish, were approved by the U.S. Geological Survey’s Leetown Science Center’s Institutional Animal Care and Use Committee (IACUC) protocol #07001. Five smallmouth bass, approximately 2 years old, were sampled from a flow-through tank at the U.S. Geological Survey Fish Health Laboratory in Kearneysville, West Virginia. Fish were placed in a lethal dosage (350 mg/L) of tricaine methanesulfonate (Tricaine-S, Syndel, Ferndale, WA) for euthanasia. An incision from the anus to operculum was made, the liver was excised, dissected into five equal pieces, and placed into fixatives consisting of NBF, ZF (Product # 171, Anatech Ltd, Battle Creek, MI), and PG (Product # 765312, QIAGEN, Valencia, CA). Pieces of liver from each fish were also placed into RNALater® (Product # AM7021, Thermo Fisher Scientific, Waltham, MA) and 95% ethyl alcohol (ETOH) to serve as controls. Samples in RNALater® were stored at 4°C for 24 hr prior to storage at -20°C and samples in ETOH were stored at room temperature (RT) until extractions were completed.

### Histological preparation and laser capture microdissection

Samples were fixed for 24 hrs, 48 hrs, seven days, and 14 days at RT for NBF and ZF. Tissues preserved in PG were removed from the PAXgene® Tissue FIX (Product # 765312, QIAGEN) after 4 hrs at RT, placed in the PAXgene® Tissue STABILIZER solution (Product # 765512, QIAGEN), and stored at 4°C for 24 hrs, 48 hrs, seven and 14 days. Tissue processing was performed on a Shandon Citadel^TM^ Tissue Processor (Thermo Fisher Scientific) as follows: 2 hrs in 65% alcohol, 1 hr in 80% alcohol, 1 hr in 95% alcohol (2x), 1 hr in 100% alcohol (3x), 1 hr in a 50/50 solution of 100% alcohol and histoclear (2x; Product # HS-200, National Diagnostics, Atlanta, GA), 2 hr in histoclear (2x), and 2 hr in paraffin (2x) at 60°C. Upon completion, tissues were embedded into paraffin wax and cooled to harden.

**Fig 1:**
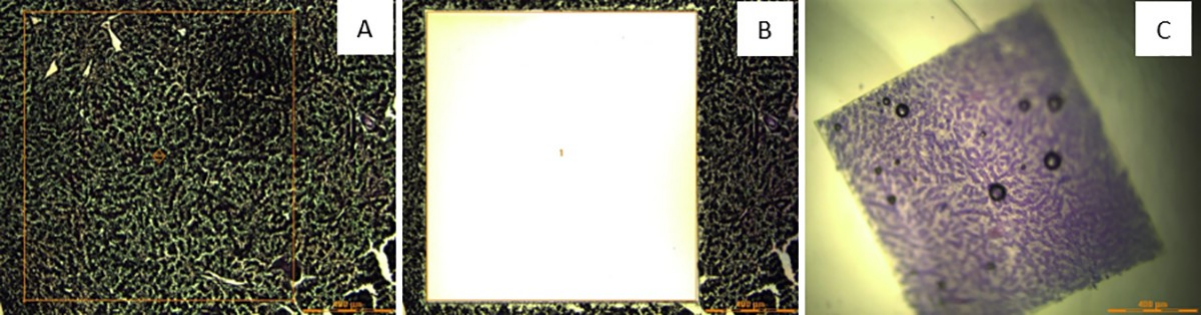
Laser capture microdissection of a smallmouth liver section. (A) Liver section prior to microdissection. (B) Liver section after microdissection. (C) Liver section floating in buffer in the cap of a microcentrifuge tub prior to nucleic acid extraction.

Tissues were cut at a thickness of 10 μm using a new, sterile razor for each sample and sections placed onto Leica Microsystems UV-sterilized polyethylene napthalate (PEN) membrane slides (Product # NC0496333, Thermo Fisher Scientific). Sterilized diethyl pyrocarbonate (DEPC, Product # 159220, Millipore Sigma, Burlington, MA) water was used in the water bath and slides were allowed to air dry after sections were placed on the PEN membrane slide. Unstained tissue sections were de-paraffinized with Anatech Ltd. Pro-Par Clearant (Product # NC9537734, Thermo Fisher Scientific) for 5 min (2x) and allowed to air dry prior to laser microdissection. Liver sections were cut at 5x magnification with a Leica LMD6500 microscope (Leica Microsystems) at a pulse rate of 55–60 nm. Sections 1,440,000 x 1,440,000 mm^2^ were cut and dropped into the cap of a sterile microcentrifuge tube by gravity (Fig 1) and subsequently extracted for RNA or DNA.

### Nucleic acid extractions and downstream analyses

For RNA purification, the E.Z.N.A.® FFPE RNA Kit (Product # R6954-01, Omega Bio-Tek, Norcross, GA) was used according to manufacturer’s protocols for the xylene extraction method. Extraction began with the addition of GPL Buffer, skipping the beginning of the protocol since the tissues were already de-paraffinized. Samples were digested with proteinase K for 30 min and eluted in 15 μl DEPC water. As part of the assay protocol, DNA contamination was removed with a step involving DNA Clearance Columns that binds genomic DNA and allows RNA to pass through the spin column. For the controls preserved in RNALater®, approximately 16–20 mg liver was extracted with an E.Z.N.A.® Total RNA Kit I (Product # R6834-02, Omega Bio-Tek) according to manufacturer’s protocols and eluted in 50 μl DEPC water. DNA contamination was also removed from these samples with the use of HiBind® RNA Mini Columns and RNase-free DNase (Product # E1091-02, Omega Bio-Tek). All samples were quantified with a Qubit® 4.0 Fluorometer (Invitrogen, Carlsbad, CA) using the Qubit® RNA HS Assay Kit (Product # Q32852, Thermo Fisher Scientific). To analyze degradation, RIN values were obtained with the Agilent RNA 6000 Pico Kit (Product # 5067–1513, Agilent Technologies, Santa Clara, CA) on an Agilent 2100 Bioanalyzer (Agilent Technologies). Following quantification, samples were stored at -80°C prior to use on the Nanostring nCounter®.

DNA was extracted with a proteinase K digestion buffer (50mM Tris-HCl, pH 8.1, 1 mM EDTA, 0.5% Tween 20, 0.1 mg/ml proteinase K) as described in Lehmann and Kreipe [45]. Each sample was extracted in 50 μl of proteinase K digestion buffer and incubated overnight at 55°C. The tubes were vortexed, centrifuged, and incubated at 95°C for 10 min to deactivate proteinase K and stored at -20°C. Approximately 17–25 mg of control liver samples preserved in 95% ETOH were extracted with a Qiagen DNeasy Blood & Tissue Kit (Product # 69506, QIAGEN) according to manufacturer’s protocols. It is worth mentioning that in initial trials for this study, the Qiagen DNeasy Blood & Tissue Kit was also used to extract DNA from LCM samples; however, no quantifiable DNA could be obtained which was why a single tube extraction method was subsequently utilized. DNA was quantified with the Qubit® dsDNA HS Assay Kit (Product # Q32851, Thermo Fisher Scientific). Samples were analyzed for mean fragment size and distribution on an Agilent 2100 Bioanalyzer with the Agilent High Sensitivity DNA Kit (Product # 5067–4626, Agilent Technologies).

For all LCM samples, the final concentration of purified RNA and DNA was standardized by dividing the total concentration by the total amount of tissue collected (μg/mm^3^). Since a greater amount of tissue was extracted from control samples, the concentration of purified RNA and DNA was standardized to the amount of tissue collected for LCM.

To assess the suitability of LCM samples for downstream molecular analyses, Sanger sequencing and the NanoString nCounter® Technology were used. For endpoint PCR, primers EF1α5F (5’-GAG CCC CCT TAC AGC CAG AAG-3’) and EF1α5R (5’-TTC ACC TCA GTG GTC AGG CA-3’) were designed with NCBI Primer BLAST [46] to amplify a 395 bp amplicon of the smallmouth bass *elongation factor 1 alpha* (*EF1α*; accession # HQ424872.1) gene. This housekeeping transcript was chosen since it has been used in other smallmouth bass studies [17, 47] and sequence data was available for both smallmouth bass and the closely related largemouth bass (accession # KT827794.1). PCR amplification was conducted under the following conditions: denaturation at 95°C for 3 min, followed by 34 cycles of 95°C for 30 s, 60°C for 30 s, and 72°C for 1 min 30 s, with a final extension at 72°C for 5 min. Each reaction consisted of 12.5 μl clear Go Taq Master Mix (Product # M7132, Promega, Madison, WI), 1.0 μl of each primer at 10μM, and 10.5 μl template for LCM samples (approximately 3–13.5 ng) and 1 μl template for ETOH samples (approximately 68–108 ng). Upon completion, samples were analyzed on an agarose gel with a 100 bp ladder. Amplicons were cleaned with a QIAquick® PCR purification kit (Product # 28104, QIAGEN) and eluted in 30 μl of Buffer EB. Purified amplicons were used as template in cycle sequencing reactions with the Applied Biosystems BigDye Terminator v3.1 Cycle Sequencing Kit (Product # 4337455, Thermo Fisher Scientific) for 25 cycles of 96°C for 1 min, 96°C for 10 s, 50°C for 5 s, and 60°C for 4 min. Cycle sequencing reactions were purified with an Agencourt CleanSEQ Kit (Product # A29151, Beckman Coulter, Brea, CA) and sequenced on an ABI 3130xl Genetic Analyzer (Applied Biosystems, Foster City, CA). Sequences were analyzed with Geneious 10.1.3 (https://www.geneious.com) and quality was assessed by the percentage of bases with a quality score of 40 or higher (Q40). NCBI BLASTn was used to determine sequence similarity to the *Micropterus* spp. *EF1α* gene (HQ424872.1 or KT827794.1).

NanoString nCounter® Technology was used with a Custom CodeSet that targeted 50 transcripts expressed in the liver of smallmouth bass as described in Hahn et al. [47]. The previous establishment and availability of this CodeSet was the reason liver was chosen as the tissue of focus in this study. The liver is also the principal organ for many chemical detoxification pathways, metabolic pathways, and the production of vitellogenin. In brief, the nCounter® platform provides the capability to quantify up to 800 RNA, DNA, or protein targets (called a CodeSet) in a multiplex fashion, providing results similar to quantitative PCR (qPCR) [44]. It is a cost-effective method to analyze specific mRNA targets, unlike RNA-sequencing which produces a vast amount of data and captures all mRNA in a sample. Sample setup for hybridizations was carried out according to manufacturer’s protocols with 25 ng of total RNA for every sample. The limit of detection (LOD) was calculated as the mean of the negative controls + 2 * the standard deviation of the negative controls and was 16 transcripts.

### Statistics

Significant differences in nucleic acid concentrations and transcript abundance between fixatives for each fixation time were determined with a nonparametric Kruskal-Wallis one-way ANOVA followed by a Dunn’s multiple comparison *post-hoc* analysis (with a Bonferroni correction) in the statistical program R [48]. Normalized transcript abundance data was used for the analysis. Transcript abundance data was normalized in nSolver Analysis Software 4.0 (Nanostring Technologies, Seattle, WA) where the geometric mean of the negative controls was subtracted to estimate background, and the normalization factor was computed from the geometric mean of the positive controls and the housekeeping transcripts. Housekeeping transcripts were log2 transformed and analyzed for stability with NormFinder [49] in R. A Kruskal-Wallis test was also used to identify differences amongst each fixative for each fixation time and the template concentration used for PCR, the Q40 score, and sequence length obtained with Sanger sequencing. Finally, Spearman’s rank correlation analyses were conducted in R to determine if the concentration of DNA samples used for PCR, sequence length, and the Q40 score were associated the number of sequences with similarity to the *Micropterus* spp. *EF1α* gene with Sanger sequencing. *P*-values ≤ 0.05 were considered statistically significant.

## Results

### Nucleic acid concentrations

**Fig 2:**
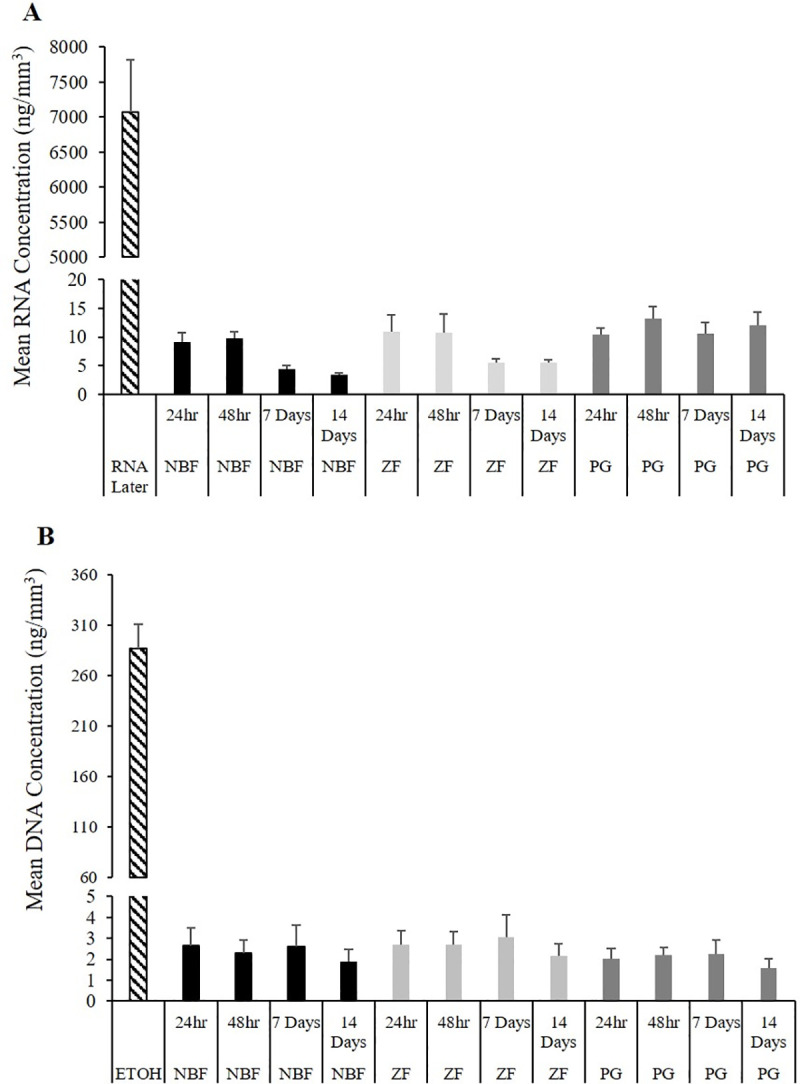
Nucleic acid auantification. (A) Mean (+ standard error) of RNA and (B) DNA concentrations (μg/mm^3^) of microdissected smallmouth bass liver samples fixed in 10% neutral buffered formalin (NBF), Z-Fix^®^ (ZF), and PAXgene® (PG) for 24 hours, 48 hours, 7 and 14 days. Samples preserved in 95% alcohol (ETOH) and RNALater® were included as controls.

Both RNA and DNA were recovered from samples of all fixatives and fixation times (Fig 2). Liver samples preserved in RNALater® had more than 800 times greater RNA concentrations than samples preserved in NBF, ZF, or PG with a mean concentration of 7,076.39 ± 737.18 ng/mm^3^ (mean ± standard error). The highest concentration of RNA from LCM samples was obtained from PG samples at 48 hr (13.25 ± 2.03 ng/mm^3^; Fig 2A). The lowest concentrations were from NBF preserved tissues at two weeks (3.40 ± 0.40 ng/mm^3^). The concentration of RNA was significantly greater in RNALater® samples than in NBF (*P*-value = 0.004) and PG (*P*-value = 0.033) at 24 hr, NBF (*P*-value = 0.005) and ZF (*P*-value = 0.019) at 48 hr, NBF (*P*-value = 0.002) and ZF (*P*-value = 0.006) at seven days, and NBF (*P*-value ≤ 0.001), ZF (*P*-value = 0.023) and PG (*P*-value = 0.023) at 14 days. Mean concentrations of RNA in samples fixed in NBF and ZF decreased at seven and 14 days, while those fixed in PG remained stable throughout the time course (Fig 2A).

The amount of DNA recovered was less than RNA, with all mean concentrations of LCM samples below 3 μg/mm^3^. Samples fixed in ETOH had more than 120 times greater concentrations of DNA than samples fixed for LCM with a mean concentration of 287.15 ± 23.76 ng/mm^3^. The concentration of DNA was significantly greater in ETOH samples than in PG at 24 hr (*P*-value = 0.001), NBF (*P*-value = 0.014) and PG (*P*-value = 0.008) at 48 hr, NBF (*P*-value = 0.016) and PG (*P*-value = 0.006) at seven days, and NBF (*P*-value = 0.026) and PG (*P*-value = 0.003) at 14 days. There was little variation in DNA concentrations over time for any of the fixatives, although for all fixatives the lowest concentration was at 14 days (Fig 2B).

**Fig 3:**
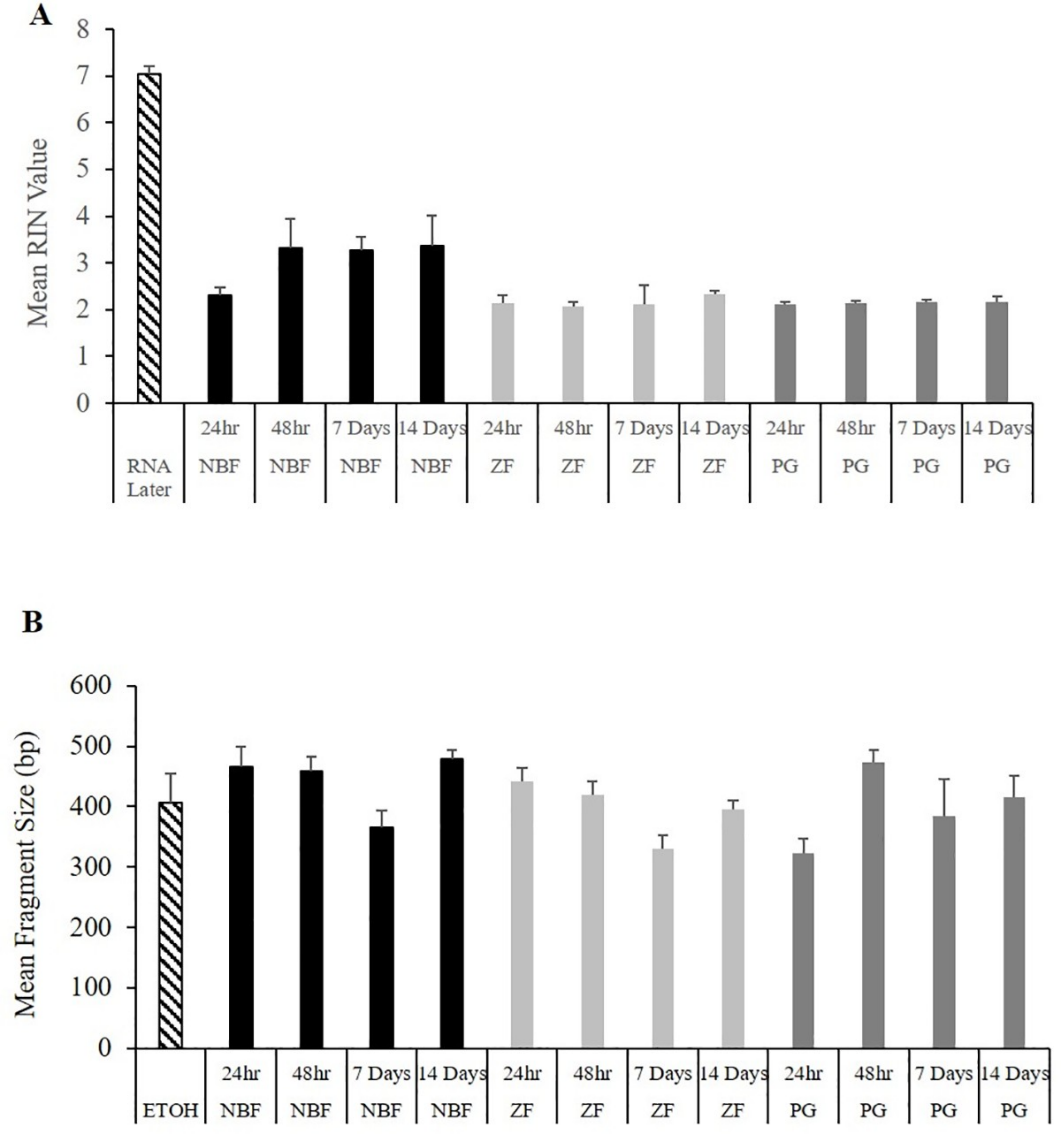
Nucleic acid quality. (A) Mean RIN values of RNA and (B) fragment size (bp) of DNA from samples fixed in 10% neutral buffered formalin (NBF), Z-Fix (ZF) and PAXgene (PG) for 24 hours, 48 hours, 7 and 14 days. Samples preserved in 95% alcohol (ETOH) and RNAlater were included as controls.

The quality of RNA varied among fixatives. Mean RIN values of samples fixed in RNALater® were at least twice as great as samples fixed in NBF, ZF, and PG. The highest RIN values for LCM samples were observed in NBF fixed tissue at 48 hrs, seven and 14 days (Fig 3A). RIN values were significantly greater in RNALater® samples than in PG (*P*-value *=* 0.005) and ZF (*P*-value = 0.005) samples at 24 hr, PG (*P*-value *=* 0.006) and ZF (*P*-value = 0.001) samples at 48 hr, PG (*P*-value = 0.001) and ZF (*P*-value = 0.007) samples at seven days, and PG (*P*-value ≤ 0.001) and ZF (*P*-value = 0.017) samples at 14 days. There were no significant differences in RIN values between LCM samples.

Mean fragment size of DNA varied over time within each fixative group but was not significantly different than samples fixed in ETOH (Fig 3B). Fragment size was only significantly greater in NBF samples than in PG samples (*P* -value = 0.026) at 24 hr.

### Downstream analyses

**Fig 4:**
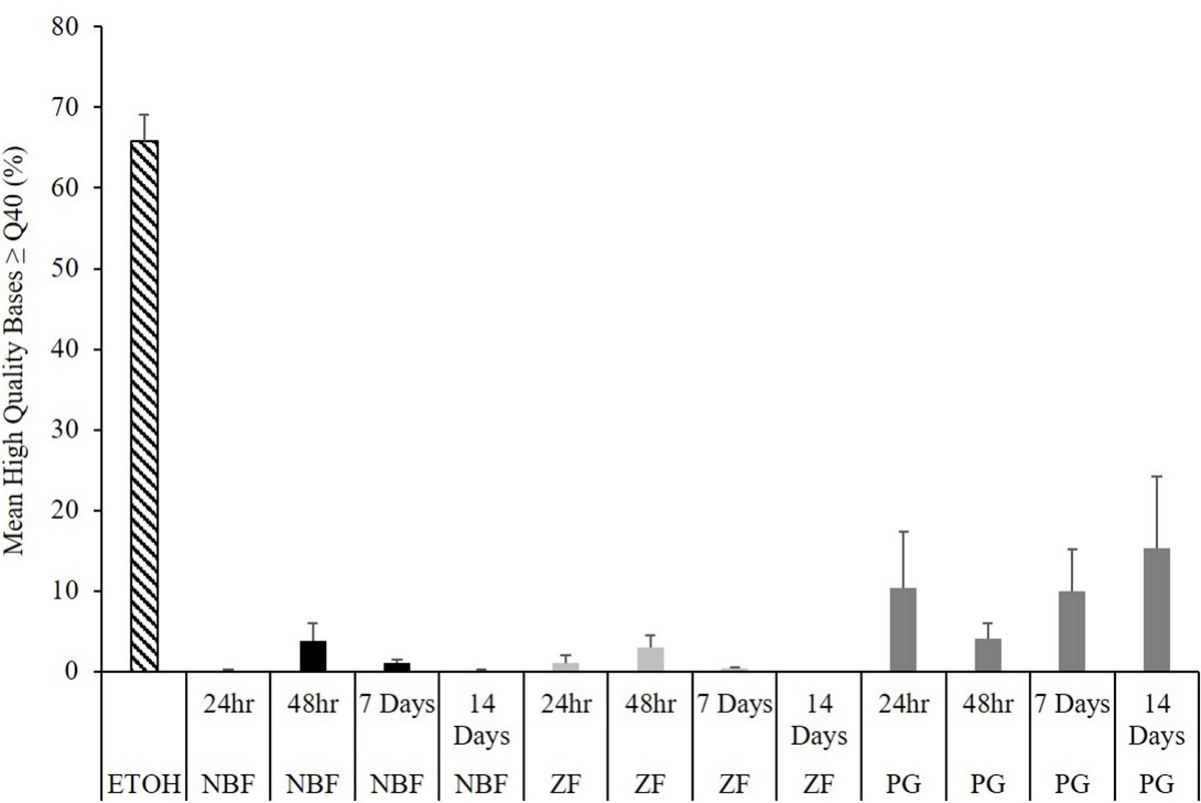
Sanger sequencing quality. Mean (+ SEM) percentage of bases with a Q40 score or above (indicative of high quality sequencing). Samples preserved in 95% ethanol (ETOH) were included as controls routinely used for DNA preservation.

End-point PCR and Sanger sequencing were successful for the amplification and sequencing of the smallmouth bass *EF1α* gene, although differences in sequencing success were apparent. Without trimming the 5’ or 3’ ends, samples preserved in ETOH produced sequences with a mean percentage of bases with a Q40 score or greater of 65.8% while samples fixed for LCM produced lower quality sequences (Fig 4). Of the samples fixed for LCM, PG preserved samples produced the highest quality sequences. At 14 days, there were no samples fixed in NBF or ZF that were successful for sequencing the *EF1α* gene. For NBF and ZF, the best quality sequences were generated by samples fixed for 48 hr, conversely PG had the lowest quality sequences from samples fixed for 48 hr (Fig 4). It should be noted that of the five PG samples sequenced at 48 hr, two samples had much lower quality sequences than the other three samples which may have contributed to the decrease in the mean percentage of high quality sequences at 48 hr. Additionally, multiple samples failed to sequence. These included one of the ETOH samples (forward and reverse sequences), three NBF 14 day samples (forward sequences), one NBF seven day sample (forward and reverse sequences), two PG seven day samples (forward sequences), one PG 14 day sample (forward sequence), one PG 24 hr sample (reverse sequence), one ZF seven day sample (reverse sequence), four ZF 14 day samples (three forward and one reverse sequence), and one ZF 48 hr sample (forward sequence). In order to calculate the percentage of sequences with similarity to the *Micropterus* spp. *EF1α* gene, failed sequences were not included (i.e. % of sequences with similarity to *Micropterus* spp. *EF1α* = # of sequences with similarity to *Micropterus* spp. *EF1α* / total # of sequences that were successfully sequenced * 100). For LCM fixed samples, NBF samples fixed for 24 and 48 hr produced the greatest percentage (10/10, 100%) of sequences with similarity to the *EF1α* gene. In ZF samples the greatest percentage of samples with similarity to the *EF1α* gene was at 24 hr (8/10, 80%) and in PG samples it was at seven days (7/8, 88%). Although PG samples produced the least amount of sequences with similarity to the *EF1α* gene at 24 and 48 hr, it produced the greatest number of sequences at seven and 14 days.

**Fig 5:**
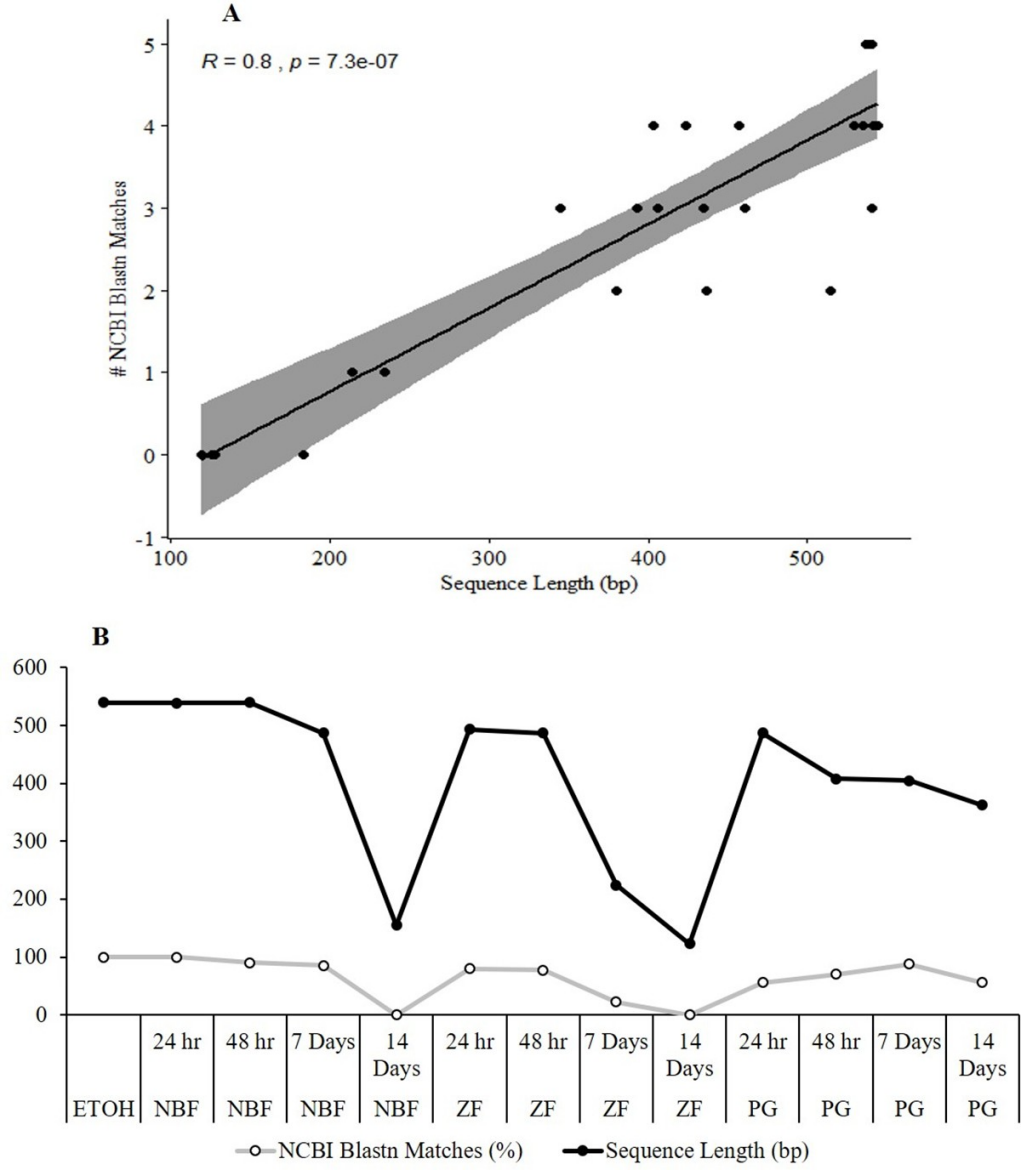
Sanger sequencing and NCBI blastn results. (A) Spearman’s rank correlation analysis of sequence length (bp) and the number of NCBI blastn matches to the *Micropterus* spp. *elongation factor 1 alpha* (*EF1α*) gene. (B) Mean sequence length (bp) of the *EF1α* gene obtained with Sanger sequencing and the percentage of sequences with similarity to the *Micropterus* spp. *EF1α* gene.

A Spearman’s rank correlation analysis with all samples revealed that sequence length (*p*-value ≤ 0.001, rho = 0.804) and template concentration (*p*-value = 0.026, rho = 0.436) were significantly associated with the number of sequences with similarity to the *EF1α* gene (Fig 5). Although PCR primers were estimated to produce an amplicon size around 395 bp, many sequences were longer than 500 bp. This could be due to the high degree of fragmentation in the samples which may have resulted in the annealing of small fragments to the original template molecules in overlapping regions [50]. Fragment length and the percentage of bases with a Q40 score or greater were not significantly correlated with the number of sequences with similarity to the *EF1α* gene. The correlations were also examined excluding the ETOH controls since the DNA concentration of the controls was significantly greater than those of many fixed samples and to examine the differences amongst the fixatives only. Sequence length remained significant (*p*-value ≤ 0.001, rho = 0.807); however, template concentration was not significant (*p*-value = 0.066, rho = 0.381). Fragment length and the percentage of bases with a Q40 score or greater remained not significantly correlated. A Kruskal-Wallis test was used to identify significant differences between fixation times and template concentration, percentage of bases with a Q40 score or greater, and sequence length for each fixative (Table 1).

**Table 1:** Kruskal-Wallis non-parametric ANOVA results: Significant differences in template concentration used in PCR and Sanger sequencing results for each fixative between fixation times.

Template Concentration			
Fixative	*p*-value	Z score	Fixation Time
NBF	No significant differences		
ZF	No significant differences		
PG	0.028	2.604	48 hr vs 14 days
% of Bases with ≥ Q40 Score			
Fixative	*p*-value	Z score	Fixation Time
NBF	0.013	2.853	48 hr vs 14 days
NBF	0.014	2.841	48 hr vs 24 hr
ZF	No significant differences		
PG	No significant differences		
Sequence Length			
Fixative	*p*-value	Z score	Fixation Time
NBF	0.001	-3.551	14 days vs 24 hr
NBF	0.002	-3.428	14 days vs 48 hr
ZF	0.005	-3.166	14 days vs 24 hr
ZF	0.018	-2.749	14 days vs 48 hr
PG	No significant differences		

Significant differences (*p*-value ≤ 0.05) in template concentration, % of bases with a Q40 score or greater, and sequence length between samples fixed in 10% neutral buffered formalin (NBF), Z-Fix® (ZF), and PAXgene (PG).

The Nanostring nCounter® results revealed multiple occurrences of samples fixed for LCM with similar or greater transcript abundance compared to RNALater® samples (S1 Table). In samples fixed for LCM there was significantly greater transcript abundance in PG samples than in NBF and/or ZF samples for one transcript at seven days and eight transcripts at 14 days. Interestingly, there were multiple significant differences in housekeeping transcript abundances between samples preserved in RNALater® and samples fixed for LCM. Mean *EF1α* transcript abundance was significantly greater in RNALater® samples than in samples fixed for LCM at all fixation times. Conversely, at seven and 14 days, *40S ribosomal protein S12* transcripts were higher in NBF and ZF samples when compared to RNALater® and *ribosomal protein L8* was higher in the PG samples (Table 2).

**Table 2:** Kruskal-Wallis non-parametric ANOVA results: Significant differences in transcript abundance between fixatives for each fixation time.

24 Hr			
Transcript Name	*p*-value	Z score	Fixatives
Elongation Factor 1 Alpha*	0.012	2.886	RNALater vs NBF
*Heat Shock Protein 70*	0.031	-2.566	RNALater vs NBF
Elongation Factor 1 Alpha*	0.014	2.833	RNALater vs PG
*Thyroid Hormone Receptor Beta*	0.005	-3.154	RNALater vs PG
48 Hr			
Transcript Name	*p*-value	Z score	Fixatives
Elongation Factor 1 Alpha*	0.003	3.261	RNALater vs PG
*Heat Shock Protein 70*	0.023	-2.673	RNALater vs NBF
Elongation Factor 1 Alpha*	0.026	2.619	RNALater vs ZF
*Heat Shock Protein 70*	0.012	-2.886	RNALater vs ZF
7 Days			
Transcript Name	*p*-value	Z score	Fixatives
40S Ribosomal Protein S12*	0.019	-2.726	RNALater vs NBF
*Arginase*	0.010	2.940	RNALater vs NBF
*Beta-catenin*	0.012	2.890	RNALater vs NBF
Elongation Factor 1 Alpha*	0.012	2.890	RNALater vs NBF
*Heat Shock Protein 70*	0.014	-2.833	RNALater vs NBF
*Transforming Growth Factor Beta*	0.024	2.647	RNALater vs NBF
40S ribosomal protein S12*	0.023	-2.673	RNALater vs ZF
*C Reactive Protein-like*	0.019	2.726	RNALater vs ZF
Elongation Factor 1 Alpha*	0.036	2.512	RNALater vs ZF
Eukaryotic Translation Initiation Factor 3D*	0.026	2.619	RNALater vs ZF
*Transforming Growth Factor Beta*	0.010	2.941	RNALater vs ZF
*Apolipoprotein 1*	0.016	2.780	RNALater vs PG
Elongation Factor 1 Alpha*	0.026	2.619	RNALater vs PG
Ribosomal Protein L8*	0.036	-2.512	RNALater vs PG
*Superoxide Dismutase*	0.010	2.940	RNALater vs PG
*Thyroid Hormone Receptor Beta*	0.012	-2.888	RNALater vs PG
*Thyroid Hormone Receptor Beta*	0.001	3.636	PG vs NBF
14 Days			
Transcript Name	*p*-value	Z score	Fixatives
40S ribosomal protein S12*	0.019	-2.726	RNALater vs NBF
*Arginase*	0.049	2.405	RNALater vs NBF
Elongation Factor 1 Alpha*	0.026	2.619	RNALater vs NBF
40S ribosomal protein S12*	0.003	-3.261	RNALater vs ZF
*Arginase*	0.031	2.566	RNALater vs ZF
*C Reactive Protein-like*	0.012	2.886	RNALater vs ZF
Elongation Factor 1 Alpha*	0.008	2.993	RNALater vs ZF
Ribosomal Protein L8*	0.031	-2.566	RNALater vs ZF
*Transforming Growth Factor Beta*	0.008	2.993	RNALater vs ZF
*Apolipoprotein 1*	0.007	3.047	RNALater vs PG
*Aryl Hydrocarbon Receptor*	0.036	-2.512	RNALater vs PG
Elongation Factor 1 Alpha*	0.049	2.405	RNALater vs PG
*Heat Shock Protein 70*	0.016	-2.780	RNALater vs PG
Ribosomal Protein L8*	0.023	-2.673	RNALater vs PG
*Aryl Hydrocarbon Receptor*	0.010	2.940	PG vs NBF
*Estrogen Receptor Beta 2*	0.023	2.673	PG vs NBF
*Hepcidin 1*	0.014	2.833	PG vs NBF
*Thyroid Hormone Receptor Beta*	0.001	3.688	PG vs NBF
*Type II Iodothyronine Deiodinase*	0.049	2.405	PG vs NBF
*Aryl Hydrocarbon Receptor*	0.031	2.566	PG vs ZF
*Hepcidin 1*	0.008	2.993	PG vs ZF
*Thyroid Hormone Receptor Beta*	0.016	2.780	PG vs ZF
*Type II Iodothyronine Deiodinase*	0.008	2.993	PG vs ZF

Significant differences (*p*-value ≤ 0.05) in transcript abundance between RNALater® samples and samples fixed in 10% neutral buffered formalin (NBF), Z-Fix® (ZF), and PAXgene (PG).

* Indicates housekeeping transcripts

Significant differences were also identified between fixation times for each fixative type used to preserve LCM samples (Table 3). All significant differences were between fixation times 24 hr and seven or 14 days and 48 hr and seven or 14 days. In some instances, samples with longer fixation times had transcripts with significantly greater transcript abundance than samples fixed for 24 or 48 hr. Once again, significant differences were identified amongst housekeeping transcripts. There were no significant differences in PG samples over time.

**Table 3:** Kruskal-Wallis non-parametric ANOVA results: Significant differences in transcript abundance between fixation times for each fixative.

Z-Fix			
Transcript Name	*p*-value	Z score	Time
40S ribosomal protein S12*	0.042	-2.459	24hr vs 14 days
	0.036	-2.512	48hr vs 14 days
*Cytochrome P450*, *family 3*, *subfamily A*	0.036	2.512	24hr vs 14 days
Elongation Factor 1 Alpha*	0.014	2.833	24hr vs 14 days
	0.016	2.780	48hr vs 14 days
Eukaryotic Translation Initiation Factor 3D*	0.036	2.512	24hr vs 14 days
Ribosomal Protein L8*	0.031	-2.566	24hr vs 14 days
*Tata-Box Binding Protein*	0.023	2.673	24hr vs 14 days
	0.042	2.459	48hr vs 14 days
*Thyroid Hormone Receptor Beta*	0.036	2.513	24hr vs 7 days
	0.016	2.781	24hr vs 14 days
	0.026	2.620	48hr vs 14 days
*Transforming Growth Factor Beta*	0.014	2.833	24hr vs 14 days
	0.023	2.673	48hr vs 14 days
NBF			
Transcript Name	*p*-value	Z score	Time
40S ribosomal protein S12*	0.049	-2.405	48hr vs 7 days
	0.019	-2.726	48hr vs 14 days
*Cytochrome P450*, *family 3*, *subfamily A*	0.036	2.512	24hr vs 14 days
*Beta-catenin*	0.042	2.459	24hr vs 7 days
Elongation Factor 1 Alpha*	0.008	2.993	48hr vs 7 days
	0.012	2.886	48hr vs 14 days
*Epoxide Hydrolase 1*	0.042	2.459	24hr vs 14 days
Eukaryotic Translation Initiation Factor 3D*	0.016	2.780	48hr vs 14 days
*Glutathione-disulfide Reductase*	0.016	2.780	24hr vs 14 days
*Hepcidin 1*	0.036	2.512	24hr vs 14 days
Ribosomal Protein L8*	0.031	-2.566	48hr vs 7 days
	0.005	-3.154	48hr vs 14 days
*Thyroid Hormone Receptor Beta*	0.026	2.619	24hr vs 7 days
	0.003	3.314	24hr vs 14 days
	0.019	2.726	48hr vs 14 days
*Transforming Growth Factor Beta*	0.006	3.100	24hr vs 7 days
	0.010	2.940	24hr vs 14 days
*Phosphoenolpyruvate Carboxykinase*	0.036	2.512	24hr vs 7 days
	0.010	2.940	48hr vs 7 days
PG			
Transcript Name	*p*-value	Z score	Time
No significant differences			

Significant differences (*p*-value ≤ 0.05) in transcript abundance by fixation time between samples fixed in 10% neutral buffered formalin (NBF), Z-Fix® (ZF), and PAXgene (PG).

* Indicates housekeeping transcripts

NormFinder results ranked the housekeeping transcripts according to stability. For all fixatives and all fixation times (including RNALater® samples) the most stable housekeeping transcript was *Ribosomal Protein L8* (stability = 0.11), followed by *EF1α* (stability = 0.21), *Eukaryotic Translation Initiation Factor 3D* (stability = 0.22), and *40S ribosomal protein S12* (stability = 0.29).

## Discussion

In this study, DNA and RNA were successfully extracted from LCM samples of smallmouth bass liver fixed in NBF, ZF, and PG. However, concentrations of nucleic acids were up to 800 and 120 times lower in samples fixed for LCM than samples preserved in RNALater® and ETOH, respectively. Overall, the recovery of RNA was greater than DNA (Fig 2). This may be attributed to the amount of DNA in a cell which can be up to five times less than the total amount of RNA [51, 52]. For LCM samples, the highest concentrations of RNA were recovered from PG fixed samples; however, at 24 and 48 hrs there was little difference among fixatives. There was also little variation in mean DNA concentrations at any time period (Fig 2).

Differences in extraction methods may have also influenced the concentration results obtained in this study. Many LCM studies utilize single tube extraction methods for DNA [34, 45, 53–55] to avoid the loss of DNA with a spin-column. In initial trials for this study, kits with spin-columns designed to extract DNA from FFPE samples were tested; however, DNA concentrations were too low to quantify, similar to other studies [34]. Additionally, a single-tube extraction method that did not use sodium dodecyl sulfate (SDS) was chosen since it acts as a PCR inhibitor [45]. Conversely, a spin column protocol worked well for RNA purification and for DNA purification from control samples fixed in ETOH. Although not significant, one finding was that the highest concentrations of DNA were extracted from samples fixed for one week. This was unexpected since longer times in formalin fixatives have been shown to affect the quality of DNA [56]. However, fragment size was the smallest for this fixation time and may have affected quantification accuracy [57], particularly in ZF and NBF samples.

Degradation of nucleic acids was also analyzed among the different treatments. For DNA, average fragment size of samples fixed for LCM ranged between 200–500 bp, similar to findings in other studies [34, 56]. These were not significantly different than ETOH fixed samples but there was variation among times for all fixatives (Fig 3B). Sanger sequencing results showed that sequence length and template concentration were significantly correlated with the number of sequences with similarity to the *Micropterus* spp. *EF1α* gene. However, when ETOH samples were removed from the correlation, only sequence length was significantly associated. Sequence length dropped off significantly at 14 days in NBF and ZF samples, but not in PG samples (Fig 5B). For Sanger sequencing, template quality, concentration, and the presence of contaminants are determining factors for success [58]. In this study, template quality and quantity were very low in the fixed samples; however, sequencing of the *Micropterus* spp. *EF1α* gene was successful except for NBF and ZF samples fixed for 14 days.

For RNA, mean RIN values ranged between 2–3.36 in the LCM samples while RNALater® samples had a mean RIN value of 7.04. Unlike other studies that have found PG fixed samples to produce better quality RNA [59], it was not evident in this study. The fragmentation of RNA (which was highest in PG and ZF samples) did not have as much of a detrimental effect on transcript abundance results as was evident for Sanger sequencing. This is likely because the probes which bind to mRNA during the nCounter® hybridization are only 100 bp long and can easily bind to fragmented targets [60], whereas the target DNA length required for PCR was close to 400 bp long.

The Nanostring nCounter® analysis revealed that RNALater® preserved samples had greater transcript abundance (for many transcripts) than samples fixed for LCM. Few studies have focused on the optimization of FFPE tissue for the Nanostring nCounter® [61, 62] and this is the first time it has been optimized with LCM fish tissue. For the Nanostring nCounter®, the recommended amount of sample input for FFPE samples is 150 ng [60]. With LCM, obtaining this amount of sample input is not always feasible, especially if the target is a single cell. In this study, a smaller quantity of RNA (25 ng) was used and proved adequate to obtain count values above the LOD for most transcripts. Veldman-Jones et al. [63] found that quantities of RNA from FFPE tissue as low as 6.25 ng did not affect highly expressed transcripts; however, lowly expressed transcripts were affected and fell below the LOD. Future studies with fish tissue fixed for LCM will be needed to determine if concentrations lower than 25 ng can be used.

Transcript abundance was significantly variable between RNALater® samples and samples fixed for LCM. In most cases, this was due to very low transcript abundance that fell below the LOD in either the RNALater® or fixed samples. However, this was not the case for the housekeeping transcripts which had counts in the range of thousands or tens of thousands. Housekeeping transcript *EF1α* was significantly higher in RNALater® samples compared to samples fixed for LCM while *40S ribosomal protein S12* and *Ribosomal Protein L8* were significantly lower. There were also significant differences in housekeeping transcript abundance in LCM fixed samples between fixation times 24 or 48 hr and seven or 14 days. Other studies have also recognized this type of variability [64–66]. Thus, it will be necessary to evaluate additional housekeeping transcripts for SMB fixed tissue samples under different fixation conditions to identify housekeepers with greater stability and less variability.

Given that sample concentration was standardized among samples, it was evident that fixation time influenced the number of transcripts that fell below the LOD. In a study on larval marine fish, fixation times longer than 48 hr significantly reduced the ability to extract mtDNA [67] and a similar result was found in this study. For NBF samples at 24hr, only one transcript was below the LOD, but this number increased to two, six, and 10 at 48 hr, seven days, and 14 days respectively (S1 Table). Z-Fix® preserved samples also saw the same effect, with five, four, eight, and nine transcripts below the LOD at 24 hr, 48 hr, seven days, and 14 days respectively. PAXgene® samples had four or five transcripts below the LOD and this was consistent over time, which highlights the tissue stability provided by this fixative.

Although the methods in this study have applicability in future LCM studies, optimization will still have to be considered when working with different tissue types, fish species, or diseased tissue. Different types of tissues have cell walls which may require specific lysis conditions to break them down in order to release the greatest amount of nucleic acids [68]. Size and composition of tissues and cells can also influence nucleic acid recovery [25]. In mammals, nucleic acid concentrations in different tissues have been shown to vary by species size and body weight [69]. Assuming this holds true for fish, the use of tissue from a large versus small fish species may require variable amounts of tissue to obtain a high enough concentration of nucleic acids for downstream molecular methods. Lastly, diseased and normal tissue can have differing concentrations of nucleic acids in the same type of cell. For mRNA, transcript abundance can vary due to transcriptome size variations in diseased tissue [70]. For future studies, it will be important to consider these types of variables; however, the methods provided here provide an applicable starting point for working with LCM tissue from fish.

To conclude, this study was successful in the extraction of small quantities of degraded nucleic acids from FFPE samples of smallmouth bass liver microdissected with LCM. Downstream sequencing and transcript quantification methods, which included Sanger sequencing and the Nanostring nCounter® technology were also effective. It was shown that PG was the best fixative for the recovery of greater concentrations of RNA and ZF was the best fixative for the greatest recovery of DNA. The use of the Nanostring nCounter® to obtain direct counts of transcripts from small quantities of FFPE tissue is especially promising, particularly since no amplification steps are required, which eliminates amplification bias. However, differences in transcript abundance among fixative and fixation times indicate comparisons of different treatments/sites must utilize similarly fixed samples to prevent fixation bias. Although ZF samples recovered greater concentrations of DNA, the findings suggest that NBF samples fixed for 24–48 hr were the best for Sanger sequencing and had few transcripts that were significantly different than the controls with the Nanostring nCounter®. While PG samples also performed well, they showed the most stability in DNA and RNA preservation over longer fixation times. The information obtained from this study will be used to perform future studies on fixed tissues from fishes with different types of tumors, pathogens, endocrine disruption, and pathological alterations to identify molecular mechanisms associated with disease. In addition, these molecular methods could potentially be applied to older, archived samples, since these sources are also valuable reservoirs of disease in fishes. However, with archived samples it will be necessary to have information on the time period between preservation of the tissue and processing and embedding into paraffin.

## Supporting information

S1 Table Nanostring nCounter results: Mean and range abundance of 50 transcripts from the liver of smallmouth bass. (CSV)
